# O-Acetyl-L-homoserine production enhanced by pathway strengthening and acetate supplementation in *Corynebacterium glutamicum*

**DOI:** 10.1186/s13068-022-02114-0

**Published:** 2022-03-14

**Authors:** Ning Li, Weizhu Zeng, Jingwen Zhou, Sha Xu

**Affiliations:** 1 National Engineering Laboratory for Cereal Fermentation Technology, Jiangnan University, 1800 Lihu Road, Wuxi, 214122 Jiangsu China; 2 State Key Laboratory of Food Science and Technology, School of Food Science and Technology, Jiangnan University, 1800 Lihu Road, Wuxi, 214122 Jiangsu China; 3 Science Center for Future Foods, Jiangnan University, 1800 Lihu Road, Wuxi, 214122 Jiangsu China; 4 Engineering Research Center of Ministry of Education on Food Synthetic Biotechnology, Jiangnan University, 1800 Lihu Road, Wuxi, 214122 Jiangsu China; 5 Jiangsu Province Engineering Research Center of Food Synthetic Biotechnology, Jiangnan University, 1800 Lihu Road, Wuxi, 214122 Jiangsu China

**Keywords:** *Corynebacterium glutamicum*, L-Homoserine acetyltransferase, O-Acetyl-L-homoserine, Acetate, Acetyl-CoA

## Abstract

**Background:**

O-Acetyl-L-homoserine (OAH) is an important potential platform chemical. However, low levels of production of OAH are greatly limiting its industrial application. Furthermore, as a common and safe amino acid-producing strain, *Corynebacterium glutamicum* has not yet achieved efficient production of OAH.

**Results:**

First, exogenous L-homoserine acetyltransferase was introduced into an L-homoserine-producing strain, resulting in the accumulation of 0.98 g/L of OAH. Second, by comparing different acetyl-CoA biosynthesis pathways and adding several feedstocks (acetate, citrate, and pantothenate), the OAH titer increased 2.3-fold to 3.2 g/L. Then, the OAH titer further increased by 62.5% when the expression of L-homoserine dehydrogenase and L-homoserine acetyltransferase was strengthened via strong promoters. Finally, the engineered strain produced 17.4 g/L of OAH in 96 h with acetate as the supplementary feedstock in a 5-L bioreactor.

**Conclusions:**

This is the first report on the efficient production of OAH with *C. glutamicum* as the chassis, which would provide a good foundation for industrial production of OAH.

**Supplementary Information:**

The online version contains supplementary material available at 10.1186/s13068-022-02114-0.

## Introduction

O-Acetyl-L-homoserine (OAH) is a potential platform chemical for the production of high-value compounds, such as L-methionine [1] and γ-butyrolactone [2]. In biological systems, neither L-homoserine nor OAH is directly involved in protein biosynthesis, but they are precursors in the biosynthesis of L-methionine and S-adenosylmethionine. L-Methionine biosynthesis is strictly regulated, and its industrial production by microbial fermentation has not been realized. The industrial production is usually carried out by enzyme conversion and chemical synthesis with L-homoserine or OAH as the precursor [3, 4]. When L-homoserine is used as the precursor, it needs to be activated by HCl before reacting with methanethiol to produce L-methionine [5]. Whereas, when OAH is selected as the precursor, it can directly react with methanethiol or 3-methylthiopropionaldehyde to form L-methionine [6]. Therefore, the production of OAH is very important for the industrial production of L-methionine.

*Escherichia coli* and *Corynebacterium glutamicum* are the most popular strains used for the production of amino acids and their derivatives, such as L-glutamate, L-lysine, L-threonine, L-serine, L-histidine [7–9], 5-aminolevulinic acid and L-ornithine [10, 11]. Compared with *E. coli*, *C. glutamicum* is a safe industrial microorganism, which is more reliable for the production of food and drug-related compounds. Reports have shown that L-homoserine and OAH have been produced efficiently in *E. coli* [12–16]. However, thus far, *C. glutamicum* has only achieved efficient production of L-homoserine [17, 18]. L-Homoserine and acetyl-CoA are the substrates for OAH biosynthesis, whereas the production of OAH in *C. glutamicum* has not been reported. L-Homoserine can be efficiently accumulated in *C. glutamicum*, indicating that OAH should also be efficiently accumulated through efficient acetyl transfer [19]. Unfortunately, the engineered *C. glutamicum* strain only efficiently accumulated L-homoserine but not OAH without knock-out of the *metX* gene in our previous studies [17, 18], suggesting that some problems need to be solved to achieve OAH accumulation. These problems may include the total enzyme activity, specific enzyme activity, heat resistance of L-homoserine acetyltransferase (MetX), and even the supply of acetyl-CoA [20–22].

Acetyl-CoA is not only a key intermediate metabolite that plays an irreplaceable role in cell growth and metabolic regulation, but also is the precursor of acetyl-CoA derivatives, whose accumulation needs to strengthen metabolic flow of acetyl-CoA biosynthesis [23, 24]. There are many biosynthetic pathways of acetyl-CoA based on different substrates, such as pyruvic acid, acetic acid, and fatty acids [25, 26]. Pyruvate forms acetyl-CoA through decarboxylation using the pyruvate dehydrogenase complex (PDH) [27]; acetate forms acetyl-CoA through the reversible Pta–Ack pathway or the irreversible ACS pathway [28–30]; fatty acids form acetyl-CoA through β-oxidation [31]. In contrast to glucose, acetate can be converted to acetyl-CoA without carbon loss. Moreover, the carbon content of acetic acid and glucose is equal, and acetate is cheaper than glucose. Therefore, at the same price, the mass of acetate is more than that of glucose [32]. In addition, strengthening the biosynthesis of CoA is another way to improve the biosynthesis of acetyl-CoA [13]. By engineering these pathways, the biosynthesis of acetyl-CoA in many microorganisms has been strengthened, resulting in the efficient accumulation of high-value acetyl-CoA derivatives [33].

In this study, an efficient OAH-producing strain was constructed via metabolic engineering based on an efficient L-homoserine-producing *C. glutamicum* strain reported in our previous study [18]. First, various L-homoserine acetyltransferase genes were individually introduced into the efficient L-homoserine-producing *C. glutamicum* strain. The best performer was chosen for further study. Then, different acetyl-CoA biosynthesis pathways were introduced to strengthen acetyl-CoA biosynthesis and explore the effects of acetyl-CoA on OAH accumulation. More importantly, different feedstocks (including acetate, citrate, and pantothenate) were added to the medium, resulting in significant increases in OAH accumulation. The production of OAH was further increased through the expression of L-homoserine dehydrogenase and L-homoserine acetyltransferase via strong promoters. These results showed that *C. glutamicum* efficiently accumulated not only L-homoserine, but also OAH. This system has great potential for the industrial production of OAH.

## Materials and methods

### Strains and plasmids

*Corynebacterium glutamicum* ATCC 13032 mutants were used to produce the target product. The plasmids pEC-XK99E and pXMJ19 were used to express the genes. The plasmid pKHAsgRNA was used for genome editing [18]. The detailed information is listed in Table 1 and Additional file 1: Table S1, respectively.

**Table 1:** Strains used in this study

Strain	Description	Source
*E. coli* JM109	Plasmid amplification	Invitrogen
*C. glutamicum* ATCC 13032	Wild type	ATCC
Cg-Hser	13032 derivative, *∆mcbR*, *∆metD*, *∆thrB*, *∆NCgl2360::P*_*sod*_*-thrA*^*S345F*^, *∆NCgl2688*, *∆metY*, *∆pck::P*_*sod*_*-aspC*, *P*_*sod*_*-pyc*^*P458S*^, *P*_*sod*_*-lysC*^*T311I*^, *P*_*sod*_*-asd*, *P*_*sod*_*-hom*^*V59A*^, *P*_*sod*_*-brnFE*, *icd*^*M1V*^, *dapA*^*M1V*^	[18]
Cg-Hser-1	Cg-Hser harboring pEC-*metX*^*r*^*_Lm*	This study
Cg-Hser-2	Cg-Hser harboring pEC-*metX_Cg*	This study
Cg-1	*∆NCgl2688::P*_*NCgl1676*_*-metX*^*r*^*_Lm* in the strain Cg-Hser	This study
Cg-2	*∆NCgl2688::P*_*sod*_*-metX*^*r*^*_Lm* in the strain Cg-Hser	This study
Cg-3	*∆NCgl2688::P*_*tuf*_*-metX*^*r*^*_Lm* in the strain Cg-Hser	This study
Cg-4	Cg-1 harboring pEC-*acs*^*L641P*^_*Se*	This study
Cg-5	Cg-1 harboring pEC-*acs*_*K12*	This study
Cg-6	Cg-1 harboring pEC-*acs*_*Pp*	This study
Cg-7	Cg-1 harboring pEC-*acs2*_*Pp*	This study
Cg-8	Cg-1 harboring pEC-*acs2*_*Sc*	This study
Cg-9	Cg-1 harboring pEC-*acsA*_*Bs*	This study
Cg-10	Cg-1 harboring pEC-*NCgl2656*-P_trc_-*NCgl2657*	This study
Cg-11	Cg-1 harboring pEC-*ackA*-P_trc_-*pta*_*K12*	This study
Cg-12	Cg-1 harboring pEC-*aceE*-P_trc_-*aceF*-P_trc_-*lpd*^*A358V*^_*K12*	This study
Cg-13	Cg-1 harboring pEC-*NCgl2167*-P_trc_-*NCgl2126*-P_trc_-*NCgl0355*	This study
Cg-14	Cg-1 harboring pXM-*metX*^*r*^_*Lm* and pEC-XK99E	This study
Cg-15	Cg-1 harboring pXM-*metX*^*r*^_*Lm* and pEC-*acs*^*L641P*^_*Se*	This study
Cg-16	Cg-1 harboring pXM-*metX*^*r*^_*Lm* and pEC-*acs2*_*Pp*	This study
Cg-17	Cg-1 harboring pEC-*thrA*^*S345F*^*_Ec*	This study
Cg-18	Cg-1 harboring pEC-*metX*^*r*^*_Lm*	This study
Cg-19	Cg-1 harboring pEC-*thrA*^*S345F*^-P_trc_-*metX*^*r*^	This study
Cg-20	Cg-1 harboring pEC-*thrA*^*S345F*^-P_tac_-*metX*^*r*^	This study
Cg-21	Cg-1 harboring pEC-*thrA*^*S345F*^-P_NCgl1676_-*metX*^*r*^	This study
Cg-22	∆*Cas9*, ∆*recET* in the strain Cg-21	This study

### Culture conditions

The culture conditions were described as our previous study [17]. *C. glutamicum* strains were grown in LBHIS medium at 30°C. For preparation of competent cells, Epo medium was used. For the production of OAH, seed medium and fermentation medium were employed with 4% inoculum. Ammonium acetate, ammonium citrate, and calcium pantothenate were added to the fermentation medium as required. For the 5-L bioreactor, the seed and fermentation media were the same as those of the shaking flask culture. The volume was 2.5 L, the rotary speed was 400 rpm, the air flow rate was 2 L/min, pH was 6.0, and the inoculum was 4%. The working concentration of isopropyl-β-D-thiogalactopyranoside (IPTG) was 0.5 mM when the seed was inoculated into the shaking flask and 5-L bioreactor. Ammonia (50% v/v) was used to adjust the pH.

### Genetic operations

Heterologous genes were codon-optimized and synthesized by GeneWiz (Suzhou, China). The genes used in this study are listed in Additional file 1: Table S2. The pKHAsgRNA was linearized with the primes pKHA2842-F and pKHA2842-R. The primers designed by Primer Premier 5 software for the construction of plasmids are listed in Additional file 1: Table S3. The DNA sequence containing promoter elements is listed in Additional file 1: Table S4.

### Analytical methods

The analytical method is the same as our previous study [17, 18]. A biophotometer D30 (Eppendorf) was used to determine OD_600_. The concentration of amino acids was measured after pre-column derivatization. The concentration of acetate and glucose were measured by HPLC using an Aminex HPX-87H column (Bio-Rad) and a refractive-index detector.

## Results and discussion

### Construction of the OAH-producing strain

Usually, the wild-type *C. glutamicum* ATCC 13032 has no capacity to accumulate OAH, even if there is a relevant biosynthetic pathway. Recently, engineered *C. glutamicum* strains have exhibited the ability to biosynthesize many amino acids including L-homoserine [18]. In biological systems, L-homoserine is the precursor of the biosynthesis of OAH [34]. Therefore, on the basis of high production of L-homoserine, a strain should be able to accumulate OAH via L-homoserine acetyltransferase. However, in our previous study, a high L-homoserine-producing strain without knock-out of the *metX* gene did not accumulate detectable OAH. This may be because the native expression of the *metX* gene was too low, and the enzyme activity was strictly regulated, resulting in the failure of acetyl transfer to L-homoserine [35].

**Fig. 1:**
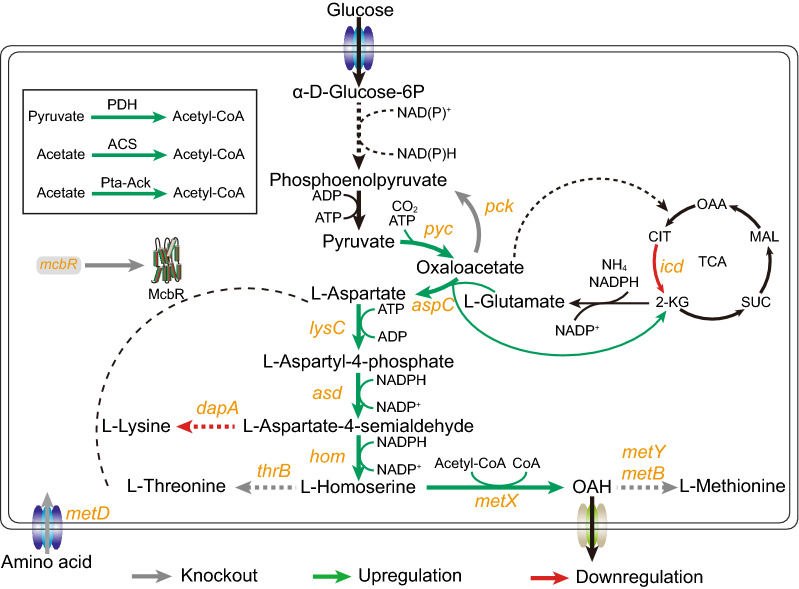
Construction of an OAH-producing strain

**Fig. 2:**
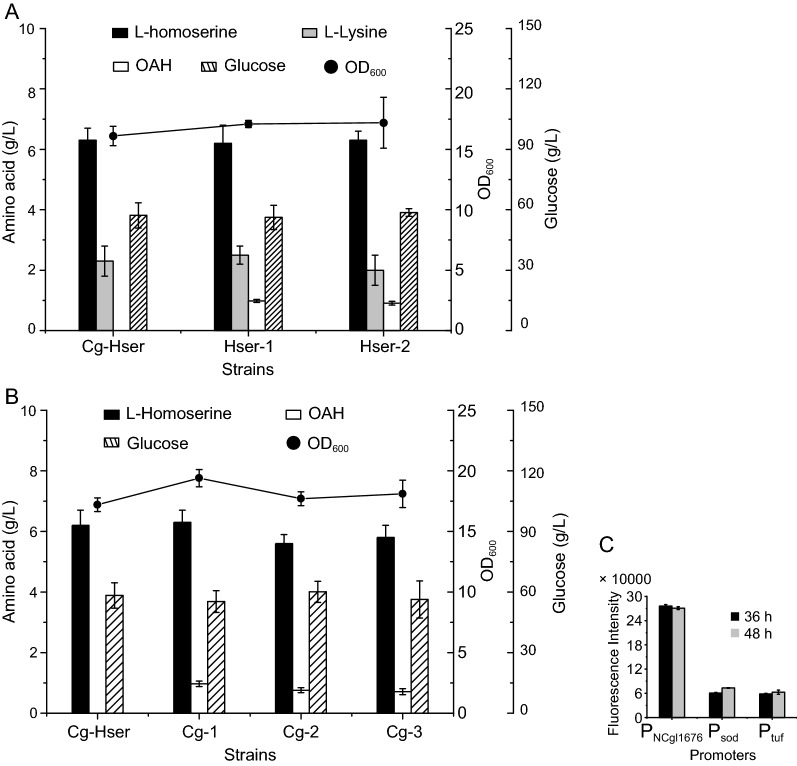
Effects of introducing different sources of *metX* on OAH accumulation. **A** Effects of different sources of *metX* on OAH accumulation; **B** the OAH production of strains with *metX*^*r*^*_Lm* gene expression under the control of different promoters; **C** the expression intensity of three promoters (P_NCgl1676_, P_sod_, P_tuf_)

In order to achieve the accumulation of OAH in *C. glutamicum*, an L-homoserine-producing strain (Cg13) was used as the starting strain, which was renamed Cg-Hser [18]. Strain Cg-Hser was derived from *C. glutamicum* ATCC 13032. In detail, some genes were successively knocked out, including *mcbR* (encoding a regulatory protein), *metD* (encoding amino acid import protein), *thrB*, *pck*, *metB*, and *metY*. The native genes including *lysC*^*T311I*^, *asd*, *hom*, *pyc*^*P458S*^, *brnFE*, and the heterologous *aspC* (from *E. coli* K12-MG1655) were upregulated though strong promoter P_sod_ in the genome. The native genes including *dapA* and *icd* were downregulated though weak start codon replacement in the genome. However, the engineered strain Cg-Hser without knock-out of the *metX* gene failed to accumulate detectable OAH. Therefore, we should strengthen the expression of L-homoserine acetyltransferase. The MetX from *Leptospira meyeri* and *C. glutamicum* ATCC 13032, whose properties have been tested in vitro in previous study [13], were chosen. Same as previous studies [17, 36], we directly expressed the *metX* genes from *Leptospira meyeri* and *C. glutamicum* ATCC 13032 by plasmid pEC-XK99E in vivo for faster screening of better performing enzymes, which generated strains Hser-1, Hser-2, respectively (Fig. 1). These engineered strains could accumulate about 0.9 g/L of OAH, and strain Hser-1 with expression of the *metX* variant gene (*metX*^*r*^) from *L. meyeri* could accumulate the highest titer of OAH (0.98 g/L) (Fig. 2A), as in *E. coli* [13].

### Introduction of different acetyl-CoA biosynthesis pathways

**Fig. 3:**
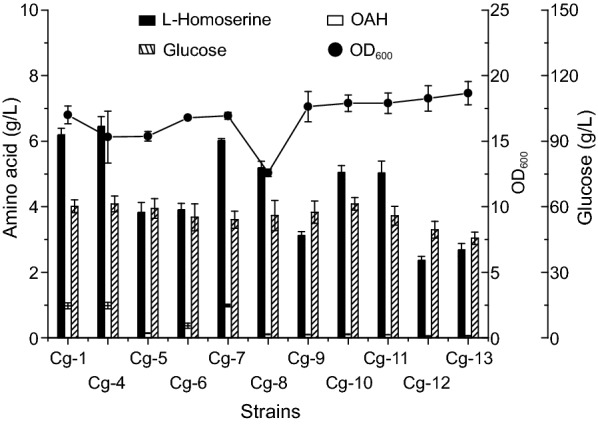
Effects of different acetyl-CoA biosynthetic pathways on OAH production

OAH did not accumulate efficiently after enhanced MetX expression though the pEC-XK99E with high copy number and strong promoter. Therefore, we turned to the supply of acetyl-CoA, which was a precursor of OAH biosynthesis in addition to L-homoserine. In order to enhance the biosynthesis of acetyl-CoA, different acetyl-CoA biosynthesis pathways were introduced. Before introducing the acetyl-CoA biosynthesis pathways, the *metX*^*r*^ from *L. meyeri* (*metX*^*r*^*_Lm*) gene was integrated into the genome of strain Cg-Hser with three strong promoters (P_NCgl1676_, P_sod_, P_tuf_) [37] (Fig. 2C), generating Cg-1, Cg-2, and Cg-3, respectively. As shown in Fig. 2B, the titers of OAH in these strains were 0.97 g/L, 0.76 g/L, 0.71 g/L, respectively. Then, we chose to upregulate the endogenous or introduce exogenous acetyl-CoA biosynthesis pathways, whose substrates were acetic acid or pyruvate, into strain Cg-1 to generate Cg-4, Cg-5, Cg-6, Cg-7, Cg-8, Cg-9, Cg-10, Cg-11, Cg-12, and Cg-13, respectively. However, the results showed that the enhancement of acetyl-CoA biosynthesis pathways did not improve the accumulation of OAH, and some of these strains even exhibited reduced accumulation (Fig. 3).

Acetyl-CoA is a direct precursor of OAH biosynthesis, and a very key intermediate metabolite and regulator in organisms [38]. Therefore, the effective supply of acetyl-CoA should be an important factor for the efficient production of OAH. Attempts were made to strengthen the acetyl-CoA biosynthesis by introducing different acetyl-CoA biosynthesis pathways, but none of them had a positive effect on OAH accumulation, and some even had negative effects. At the same time, L-homoserine production was diminished, indicating that the introduction of acetyl-CoA biosynthesis pathways led to the reduction of metabolic flow in the direction of biosynthesis of L-homoserine and OAH. The acetyl-CoA biosynthesis pathway (derived from *S. enterica* and *P. putida*) with acetate as its substrate had no positive or negative effects on OAH accumulation, probably because this pathway did not compete with L-aspartate family amino acids for pyruvate [39]. These results were very different from those in *E. coli*, in which acetyl-CoA biosynthesis was directly improved to promote the efficient accumulation of OAH on the basis of the efficient production of L-homoserine [13]. This suggested that, as a branch substance, the rational distribution of pyruvate was very important when it formed two direct substrates of the target product in *C. glutamicum*. Therefore, the factors limiting the further accumulation of OAH in *C. glutamicum* needed to be further explored.

### Effects of several feedstocks on OAH accumulation

**Fig. 4:**
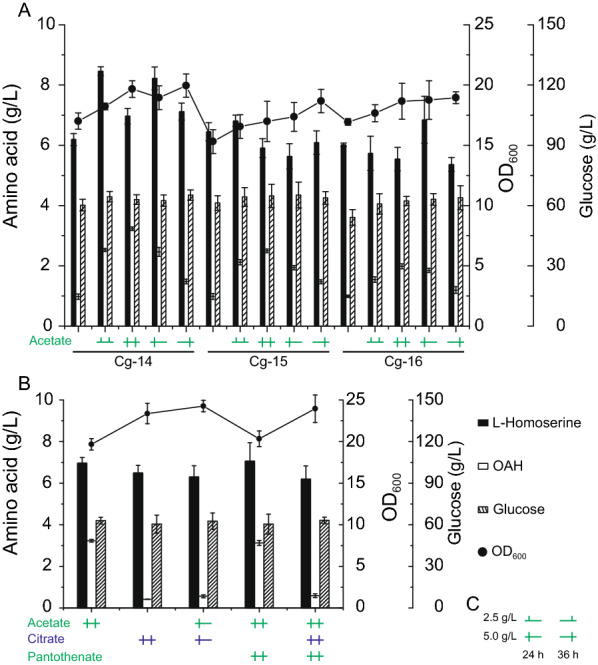
Effects of several feedstocks on OAH accumulation. **A** Effects of acetate on OAH production. **B** Effects of citrate and pantothenate on the OAH production of strain Cg-14. **C** The addition methods of the several feedstocks

The introduction of the exogenous acetate derived acetyl-CoA biosynthesis pathway failed to improve the accumulation of OAH. We speculated that this might be because there was no acetate available as a substrate for the biosynthesis of acetyl-CoA. Although some *C. glutamicum* strains can accumulate acetate [40], an analysis of the fermentation broth components found that all engineered strains in this study could not accumulate acetate under the culture conditions of this study. Therefore, to improve the biosynthesis efficiency of acetyl-CoA from acetate, it was necessary to add acetate to the culture medium. To avoid an adverse effect on cell growth caused by a sudden drop in pH, ammonium acetate was selected as the additive instead of acetic acid. Previous studies showed that L-homoserine could accumulate a high titer only after fermentation for 24 h [17, 18]. To convert the added acetate into acetyl-CoA that could be used for acetylation of L-homoserine, 2.5 g/L of acetate was added at 24 h and 36 h. The results showed that the OAH titer of the engineered strains (Cg-4, Cg-7) did not increase when the *metX*^*r*^*_Lm* gene was only integrated into the genome. Whereas, the OAH titer increased significantly when the *metX*^*r*^*_Lm* gene was overexpressed via the plasmid. As shown in Fig. 4A, the OAH titers of strains Cg-15 and Cg-16 were 2.1 g/L and 1.5 g/L, respectively. At this time, acetate was fully utilized, and the consumption of glucose did not change much, but the OD_600_ of the strains increased. Interestingly, the OAH titer of strain Cg-14, which only expressed the *metX*^*r*^*_Lm* gene without introducing acetyl-CoA biosynthase, was higher after addition of acetate, up to 2.5 g/L.

Acetate was completely consumed, indicating that its addition may be the limiting factor in the OAH accumulation. Therefore, five feeding methods of acetate were chosen to study the effects on the OAH titers. The OAH titer of Cg-14 was the highest (3.2 g/L) after 5.0 g/L of acetate was added at 24 h and 36 h, and this was 28% higher than when 2.5 g/L of acetate was added at 24 h and 36 h (Fig. 4A). However, when the addition of acetate was increased by 100%, the OAH titer increased by only 28%. Pantothenate is the precursor of CoA, which is the precursor of Acetyl-CoA. Acetyl-CoA is the competitive precursor for the biosynthesis of citric acid and OAH. To enhance the supply of acetyl-CoA for OAH biosynthesis and reduce the consumption of acetyl-CoA for citric acid biosynthesis in TCA cycle, pantothenate and citrate were also added to the culture medium. Strain Cg-14 was again employed and five feeding methods were chosen. The results showed that the citrate feeding significantly increased the biomass of the strain, but the OAH titer decreased sharply to about 0.5 g/L, indicating that citrate was not conducive to OAH accumulation. Different from citrate, the pantothenate feeding did not affect the OAH accumulation (Fig. 4B).

*Corynebacterium glutamicum* has an acetic acid biosynthesis pathway and the ability to accumulate acetic acid, but this ability is different under different culture conditions [41]. Under the culture conditions of this study, the strains could not accumulate acetic acid. Therefore, acetate needed to be added to make the introduced acetyl-CoA synthase function [42]. After acetate feeding, the titers of L-homoserine and OAH were both increased. Unexpectedly, the titer of OAH decreased after the introduction of acetyl-CoA biosynthase, indicating that the strains had a sufficient native capacity of acetate acetylation [43], and overexpression could cause a metabolic burden. Acetyl-CoA condenses with oxaloacetic acid by citrate synthase to form citric acid and then enters the TCA cycle. The citric acid feeding can improve the efficiency of the TCA cycle, but it leads to a sharp decrease in OAH accumulation, which may be because the ability to biosynthesize acetyl-CoA was strongly inhibited by citric acid, resulting in insufficient supply for L-homoserine acetylation even though acetate was added [44].

### Enhanced expression of L-homoserine acetyltransferase

**Fig. 5:**
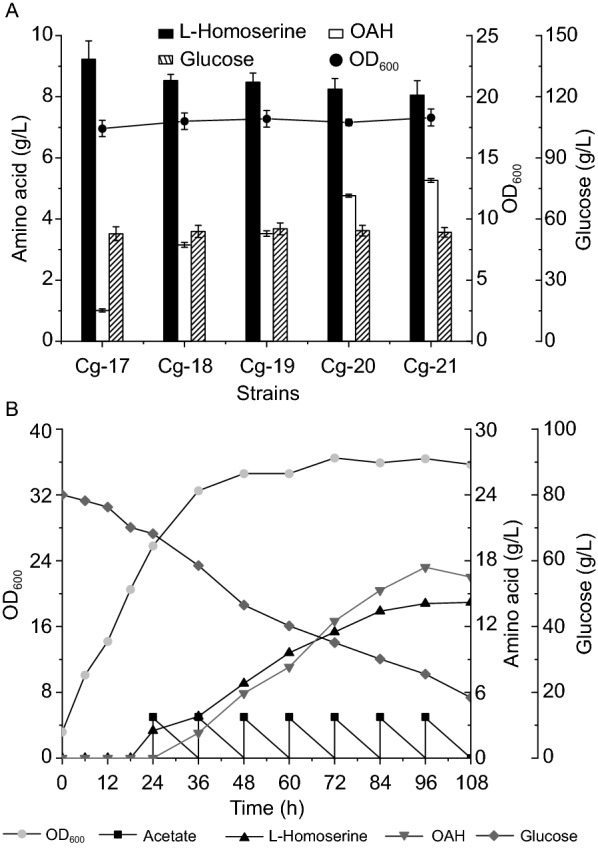
Effects of expression of *thrA*^*S345F*^ and *metX*^*r*^ genes with strong promoters on OAH production. **A** Effects of plasmid expression of *thrA*^*S345F*^ and *metX*^*r*^ genes with different strong promoters on OAH production; **B** OAH production in a 5-L bioreactor

In addition to acetate feeding, the introduction of different exogenous acetyl-CoA biosynthesis pathways and the citrate and pantothenate feeding failed to improve OAH accumulation. We speculated that the expression level of pathway enzymes would become the main limiting factor for the OAH accumulation after acetate feeding. Therefore, the *thrA*^*S345F*^ gene (encoding bifunctional L-aspartokinase and L-homoserine dehydrogenase) from *E. coli* and the *metX*^*r*^*_Lm* was overexpressed by pEC-XK99E in strain Cg-1, resulting in strains Cg-17 and Cg-18, respectively. However, the OAH titer only reached 1.1 g/L and 3.2 g/L when 5.0 g/L of acetate was added at 24 h and 36 h, respectively (Fig. 5A). In order to further enhance OAH accumulation, we used three strong promoters (P_trc_, P_tac_, P_NCgl1676_) to control the expression of the *metX*^*r*^ gene from *L. meyeri* after overexpression of the *thrA*^*S345F*^ gene in plasmid pEC-XK99E [37], resulting in strains Cg-19, Cg-20, and Cg-21, respectively. As shown in Fig. 5A, the L-homoserine titers of the strains were 8.5 g/L, 8.2 g/L, and 8.0 g/L, respectively; and the OAH titers were 3.5 g/L, 4.8 g/L, and 5.2 g/L, which increased by 9.4%, 50.0%, and 62.5% compared with the control strain Cg-14, when 5.0 g/L of acetate was added at 24 h and 36 h, respectively. These results showed that the supply of acetyl-CoA was improved after the addition of acetate, and the OAH titer could be increased through the enhanced expression of L-homoserine acetyltransferase.

### The 5-L bioreactor for OAH production

A high concentration of acetate has a strong inhibitory effect on strain growth, and the pH of the fermentation process cannot be controlled in the shaking flask. Therefore, in order to explore the potential of acetate as a feedstock and improve OAH production, a 5-L bioreactor was used to carry out further experiments. As L-homoserine is the precursor of OAH biosynthesis, we chose the same conditions as in the previous L-homoserine production for OAH production. Before that, the *cas9* and *recET* genes in the genome of strain Cg-21 were deleted, generating strain Cg-22. According to the above experiments, acetate (20% v/v) was added at 24 h to reach the concentration of 5 g/L, then it was added every 12 h. As shown in Fig. 5B, the OAH titer of strain Cg-22 reached 17.4 g/L after 96 h, which was the highest titer, with 14.1 g/L of L-homoserine. These results suggested that acetate could improve the conversion of L-homoserine and the titer of OAH. This will provide a good basis for the industrial production of OAH.

## Conclusion

In this study, exogenous L-homoserine acetyltransferase was introduced into an L-homoserine-producing strain. Then, the effects of the introduction of the acetyl-CoA biosynthesis pathway and the addition of various feedstocks on the OAH biosynthesis were compared, resulting in improving OAH production to 3.2 g/L. Through the strong promoters to control the expression of L-homoserine acetyltransferase, the titer of OAH increased to 5.2 g/L. Finally, the OAH titer reached 17.4 g/L at 96 h in a 5-L bioreactor. This is the first time to achieve efficient production of OAH in *C. glutamicum*.

## Supplementary Information

**Additional file 1: Table S1.** The constructed plasmids. **Table S2.** The used genes. **Table S3.** The used primers. **Table S4.** The DNA sequence of promoter elements.

## Data Availability

All data generated or analyzed during this study are included in this published article and its additional files.

## Abbreviations

OAH: O-Acetyl-L-homoserine
MetX: L-Homoserine acetyltransferase
PDH: Pyruvate dehydrogenase complex
*thrB*: L-Homoserine kinase gene
*pck*: Phosphoenolpyruvate carboxykinase gene
*metB*: Cystathionine gamma-synthase gene
*metY*: O-Acetylhomoserine sulfhydrylase gene
*lysC*: Aspartokinases gene
*asd*: Aspartate-semialdehyde dehydrogenase
*hom*: L-Homoserine dehydrogenase
*pyc*: Pyruvate carboxylase
*brnFE*: Branched-chain amino acid permease gene cluster
*aspC*: Aspartate transaminase gene
*icd*: Isocitrate dehydrogenase gene
*dapA*: 4-Hydroxy-tetrahydrodipicolinate synthase gene
