# Supplementary material for: O-Acetyl-L-homoserine production enhanced by pathway strengthening and acetate supplementation in Corynebacterium glutamicum
Source: Biotechnol Biofuels Bioprod. 2022 Mar 14;15:27. doi: 10.1186/s13068-022-02114-0 (PMC8922893; doi:10.1186/s13068-022-02114-0)
Supplement: Supplementary file 1 — Additional file 1: Table S1. The constructed plasmids. Table S2. The used genes. Table S3. The used primers. Table S4. The DNA sequence of promoter elements. [file 13068_2022_2114_MOESM1_ESM.pdf]

Supporting Information

O-Acetyl-L-homoserine production enhanced by pathway strengthening and acetate supplementation in *Corynebacterium glutamicum*

Ning Li[1,2,3], Weizhu Zeng[1,3], Jingwen Zhou[1,3,4,5], Sha Xu[1,3*]

[1] National Engineering Laboratory for Cereal Fermentation Technology, Jiangnan University, 1800 Lihu Road, Wuxi, Jiangsu 214122, China;

[2] State Key Laboratory of Food Science and Technology, School of Food Science and Technology, Jiangnan University, 1800 Lihu Road, Wuxi, Jiangsu 214122, China;

[3] Science Center for Future Foods, Jiangnan University, 1800 Lihu Road, Wuxi, Jiangsu 214122, China;

[4] Engineering Research Center of Ministry of Education on Food Synthetic Biotechnology, Jiangnan University, 1800 Lihu Road, Wuxi, Jiangsu 214122, China;

[5] Jiangsu Province Engineering Research Center of Food Synthetic Biotechnology, Jiangnan University, 1800 Lihu Road, Wuxi, Jiangsu 214122, China.

[*]Corresponding author

Sha Xu

Mailing address: Science Center for Future Foods, Jiangnan University, 1800 Lihu Road, Wuxi, Jiangsu 214122, China.

Phone: +86-510-85914371, Fax: +86-510-85914371

E-mail: xusha1984@jiangnan.edu.cn

Supporting tables

Table S1. Plasmids used in this study.

Plasmid	Description	Source
pEC-XK99E	IPTG-inducible P_{trc} promoter, Km^r	[1]
pXMJ19	IPTG-inducible P_{tac} promoter, Cm^r	[2]
pEC-*thrA*^{S345F}*_Ec*	pEC-XK99E harboring *thrA*^{S345F}	[3]
pEC-*metX_Go*	pEC-XK99E carrying *metX* gene from *G. oxydans* H24	This study
pEC-*metX_Pa*	pEC-XK99E carrying *metX* gene from *P. aeruginosa*	This study
pEC-*metX_Pp*	pEC-XK99E carrying *metX* gene from *P. pastoris* GS115	This study
pEC-*metX2_Sc*	pEC-XK99E carrying *metX2* gene from *S. cerevisiae* S288C	This study
pEC-*metX*^{r}*_Lm*	pEC-XK99E carrying *metX*^{r} gene from *L. meyeri*	This study
pEC-*metX_Cg*	pEC-XK99E carrying *metX* gene from *C. glutamicum* ATCC 13032	This study
pKHA-$P_{NCgl1676}$-*metX*^{r}*_Lm*	Integrated expression of *metX*^{r}*_Lm* gene under the control of promoter $P_{NCgl1676}$	This study
pKHA-P_{sod}-*metX*^{r}*_Lm*	Integrated expression of *metX*^{r}*_Lm* gene under the control of promoter P_{sod}	This study
pKHA-P_{tuf}-*metX*^{r}*_Lm*	Integrated expression of *metX*^{r}*_Lm* gene under the control of promoter P_{tuf}	This study

pXM-*metX_Cg*	pEC-XK99E carrying *metX* gene from *C. glutamicum* ATCC 13032	This study
pXM-*metX*^{r}*_Lm*	pXMJ19 carrying *metX*^{r}*_Lm* gene from *L. meyeri*	This study
pEC-*acs*^{L641P}*_Se*	pEC-XK99E carrying *acs*^{L641P} gene from *Salmonella enterica*	This study
pEC-*acs_K12*	pEC-XK99E carrying *acs* gene from *E. coli* K12-MG1655	This study
pEC-*acs_Pp*	pEC-XK99E carrying *acs* gene from *Pseudomonas putida*	This study
pEC-*acs2_Pp*	pEC-XK99E carrying *acs2* gene from *P. putida*	This study
pEC-*acs2_Sc*	pEC-XK99E carrying *acs2* gene from *S. cerevisiae* S288C	This study
pEC-*acsA_Bs*	pEC-XK99E carrying *acsA* gene from *Bacillus subtilis* 168	This study
pEC-*NCgl2656*-P$_{trc}$-*NCgl2657*	pEC-XK99E carrying *pta* and *pck* genes from *C. glutamicum* ATCC 13032	This study
pEC-*ackA*-P$_{trc}$-*pta_K12*	pEC-XK99E carrying *pta* and *pck* genes from *E. coli* K12-MG1655	This study
pEC-*aceE*-P$_{trc}$-*aceF*-P$_{trc}$-*lpd*^{A358V}*_K12*	pEC-XK99E carrying *aceE*, *aceF* and *lpd*^{A358V} genes from *E. coli* K12-MG1655	This study
pEC-*NCgl2167*-P$_{trc}$-*NCgl2126*-P$_{trc}$-*NCgl0355*	pEC-XK99E carrying *aceE*, *aceF* and *lpd* genes from *C. glutamicum* ATCC 13032	This study

pEC-*thrA*^{S345F}-P$_{trc}$-*metX*^{r}	pEC-XK99E carrying *thrA*^{S345F} and *metX*^{r}, *metX*^{r} under the control of P$_{trc}$	This study
pEC-*thrA*^{S345F}-P$_{tac}$-*metX*^{r}	pEC-XK99E carrying *thrA*^{S345F} and *metX*^{r}, *metX*^{r} under the control of P$_{tac}$	This study
pEC-*thrA*^{S345F}-P$_{NCgl1676}$-*metX*^{r}	pEC-XK99E carrying *thrA*^{S345F} and *metX*^{r}, *metX*^{r} under the control of P$_{NCgl1676}$	This study

Table S2. The genes used in this study.

**Gene**	**Sequence (5'-3')**
thrA^{S345F}	atgcgagtgttgaagttcggcggtacatcagtggcaaatgcagaacgttttctgcgtgttgccgatattct<br>ggaaagcaatgccaggcaggggcaggtggccaccgtcctctctgcccccgccaaaatcaccaacca<br>cctggtggcgatgattgaaaaaaccattagcggccaggatgctttacccaatatcagcgatgccgaac<br>gtatttttgccgaacttttgacgggactcgccgccgcccagccggggttcccgctggcgcaattgaaaa<br>ctttcgtcgatcaggaatttgcccaaataaaacatgtcctgcatggcattagtttgttggggcagtgcccg<br>gatagcatcaacgctgcgctgatttgccgtggcgagaaaatgtcgatcgccattatggccggcgtatta<br>gaagcgcgcggtcacaacgttactgttatcgatccggtcgaaaaactgctggcagtggggcattacct<br>cgaatctaccgtcgatattgctgagtccacccgccgtattgcggcaagccgcattccggctgatcacat<br>ggtgctgatggcaggtttcaccgccggtaatgaaaaaggcgaactggtggtgcttggacgcaacggtt<br>ccgactactctgctgcggtgctggctgcctgtttacgcgccgattgttgcgagatttggacggacgttga<br>cggggtctatacctgcgacccgcgtcaggtgcccgatgcgaggttgttgaagtcgatgtcctaccagg<br>aagcgatggagctttcctacttcggcgctaaagttcttcacccccgcaccattacccccatcgcccagtt<br>ccagatcccttgcctgattaaaaataccggaaatcctcaagcaccaggtacgctcattggtgccagccg<br>tgatgaagacgaattaccggtcaagggcatttccaatctgaataacatggcaatgttcagcgtttctggtc<br>cggggatgaaagggatggtcggcatggcggcgcgcgtctttgcagcgatgtcacgcgcccgtattttc

	gtggtgctgattacgcaatcatcttccgaatacagcatcagtttctgcgttccacaaagcgactgtgtgcg<br>agctgaacgggcaatgcaggaagagttctacctggaactgaaagaaggcttactggagccgctggca<br>gtgacggaacggctggccattatctcggtggtaggtgatggtatgcgcaccttgcgtgggatctcggc<br>gaaattctttgccgcactggcccgcgccaatatcaacattgtcgccattgctcagggatcttctgaacgct<br>caatctctgtcgtggtaaataacgatgatgcgaccactggcgtgcgcgttactcatcagatgctgttcaat<br>accgatcaggttatcgaagtgtttgtgattggcgtcggtggcgttggcggtgcgctgctggagcaactg<br>aagcgtcagcaaagctggctgaagaataaacatatcgacttacgtgtctgcggtgttgccaactcgaag<br>gctctgctcaccaatgtacatggccttaatctggaaaactggcaggaagaactggcgcaagccaaaga<br>gccgtttaatctcgggcgcttaattcgcctcgtgaaagaatatcatctgctgaacccggtcattgttgact<br>gcacttccagccaggcagtggcggatcaatatgccgacttcctgcgcgaaggtttccacgttgtcacgc<br>cgaacaaaaaggccaacacctcgtcgatggattactaccatcagttgcgttatgcggcggaaaaatcg<br>cggcgtaaattcctctatgacaccaacgttggggctggattaccggttattgagaacctgcaaaatctgc<br>tcaatgcaggtgatgaattgatgaagttctccggcattctttctggttcgctttcttatatcttcggcaagtta<br>gacgaaggcatgagtttctccgaggcgaccacgctggcgcgggaaatgggttataccgaaccggac<br>ccgcgagatgatctttctggtatggatgtggcgcgtaaactattgattctcgctcgtgaaacgggacgtg<br>aactggagctggcggatattgaaattgaacctgtgctgcccgcagagtttaacgccgagggtgatgttg<br>ccgcttttatggcgaatctgtcacaactcgacgatctctttgccgcgcgcgtggcgaaggcccgtgatg<br>aaggaaaagttttgcgctatgttggcaatattgatgaagatggcgtctgccgcgtgaagattgccgaagt<br>ggatggtaatgatccgctgttcaaagtgaaaaatggcgaaaacgccctggccttctatagccactattat<br>cagccgctgccgttggtactgcgcggatatggtgcgggcaatgacgttacagctgccggtgtctttgct<br>gatctgctacgtaccctctcatggaagttaggagtctga
metX^r	atgccaacctccgaacagaacgagttctcccacggttccgtgggcgtggtgtacacccagtccattcgt<br>ttcgagtccctcactctggaaggtggcgaaaccatcacccctctggagatcgcttacgaaacctacggc<br>accctcaacgaaaagaaggacaacgctattctggtgtgccacgctctgtctggcgacgcacacgcag<br>ctggcttccacgaaggtgacaagcgtccgggctggtgggactattacatcggcccgggcaagtctttc

gacaccaatcgttacttcatcatctcctccaatgtcattggcggttgcaagggttcctctggtcctctcact
atcaacggcaagaacggcaagccattccagtccaccttcccattcgtgtccatcggtgacatggtcaat
gcacaagaaaagctcatctcccatctgggtatccacaagctgttcgctgtggctggcggctctatgggc
ggtatgcaagcactgcagtggtctgtggcttatccagaccgtctgaagaactgcatcgtgatcgcttcct
cttccgagcactccgctcagcagatcgcattcaacgaggtcggccgccaagcaattctgtccgaccca
aattggaaccaaggcctctacacccaagaaaaccgcccatctaagggtctcgcactggcacgcatgat
gggtcacatcacctacctctccgacgaggcaatgcgcgagaaattcggccgtaagcctccaaaaggc
aatatccagtccaccgacttcgctgtcggttcctacctcatctaccaaggcgagtcctttgtcgaccgctt
cgacgcaaattcctacatctacgtcaccaaggctctggaccatttctctctgggtactggcaaggagctg
accaaggtcctcgctaaagtgcgctgtcgttttctggtggtcgcatatacctctgactggctgtacccacc
ttaccagtccgaagagatcgtcaagtctctggaggtcaacgcagtgccagtgtccttcgtcgagctgaa
taacccagctggccatgactcctttctgctgccatccgaacagcaagattccattctccgtgactttctgtc
ctccaccgatgaaggcgtgttcctgtaa

Table S3. The primers used in this study.

Primer	Primer sequence (5'-3')	Function
metX_H24-F	CAGACCATGGAATTCAGAAGGAGACTAGTAATGGATCGCAGTGTGTCTCCTTC	pEC-*metX_Go*
metX_H24-R	GTCGACTCTAGAGGATCCTCAGAGTGCCTCCACCGTGG	
metX_Pp-F	CAGACCATGGAATTCAGAAGGAGACTAGTAATGCAGGAGGTTCATGAGCCCGAA	pEC-*metX_Pp*
metX_Pp-R	GTCGACTCTAGAGGATCCTTACCACG	

	ACGTCGGATCCTCA	
metX_Pa-F	CAGACCATGGAATTCAGAAGGAGACTAGTAATGCCCACAGTCTTCCCCG	pEC-*metX_Pa*
metX_Pa-R	GTCGACTCTAGAGGATCCTCACACGCTGATGCGGTTCAT	
metX_Sc-F	CAGACCATGGAATTCAGAAGGAGACTAGTAATGTCGCATACTTTAAAATCGAAAACG	pEC-*metX2_Sc*
metX_Sc-R	GTCGACTCTAGAGGATCCCTACCAGTTGGTAACTTCTTCGGCC	
metX^{r}_Lm-F	CAGACCATGGAATTCAGAAGGAGACTAGTAATGCCAACCTCCGAACAGAAC	pEC-*metX*^{r}*_Lm*
metX^{r}_Lm-R	CAGGTCGACTCTAGAGGATCCTTACAGGAACACGCCTTCATCG	
metX_Cg-F	CAGACCATGGAATTCAGAAGGAGACTAGTAATGCCCACCCTCGCGCCTT	pEC-*metX_Cg*
metX_Cg-R	CAGGTCGACTCTAGAGGATCCTTAGATGTAGAACTCGATGTAGGTCGAAGG	
pKHA2842-F	GATCCCCGGGTACCGAGCT	pKHAsgRNA linearized
pKHA2842-R	ACCTACTCCTACGACCCCGAA	
NCgl2688-D-F	AGCAGAATCCAACGCAGCAGAAC	Integrated expression of *metX*^{r} gene
NCgl2688-D-R	TTCGGGGTCGTAGGAGTAGGTGGACAAGGCGGTGACCAAGTAG	

metX^{r}_Lm-1676-F	ATTATATGATTGGTTAGGACTATGGAC	
	ATGATGCCAACCTCCGAACAGAAC	
metX^{r}_Lm-sod-F	GAAACCTACGAAAGGATTTTTACCC	
	ATGCCAACCTCCGAACAGAAC	
metX^{r}_Lm-tuf-F	CACGAAGTCCAGGAGGACATACAATG	
	CCAACCTCCGAACAGAAC	
metX^{r}_Lm-R2	GTTCTGCTGCGTTGGATTCTGCTTTAC	
	AGGAACACGCCTTCATCGG	
$P_{NCgl1676}$-F	ATGCGACAGTACTTTTCATTAAGCCTA	
	A	
$P_{NCgl1676}$-R	CATGTCCATAGTCCTAACCAATCATAT	
	AAT	
P_{sod}-F	TAGCTGCCAATTATTCCGGGC	
P_{sod}-R	GGGTAAAAAATCCTTTCGTAGGTTTC	
P_{tuf}-F	GTAGGGTAAGTGGGGTAGCGG	
P_{tuf}-R	TGTATGTCCTCCTGGACTTCGTG	
NCgl2688-U-F	CCCCGATCACACTAGTGGAGTAGCTAT	Homologous arm
	GGAAGACGATCTCAGTGCTGC	
NCgl2688-1676-R	TTAGGCTTAATGAAAAGTACTGTCGCA	
	TGACGCCACGCAGGGTTAATAGG	
NCgl2688-sod-R	GCCCGGAATAATTGGCAGCTAGACGC	
	CACGCAGGGTTAATAGG	
NCgl2688-tuf-R	CCGCTACCCCACTTACCCTACGACGCC	
	ACGCAGGGTTAATAGG	
P_{glyA}-F	AGCTACTCCACTAGTGTGATCGGGG	

P_{glyA}-R	GCCCACAAGCATAGACCGCC	
2688-sgRNA-F	GTCTATGCTTGTGGGC*ACCCACAGTAACTGTGCAAG*GTTTTAGAGCTAGAAATAGCAAG	
2688-sgRNA -R	GAGCTCGGTACCCGGGGATCGTAGGGATAACAGGGTAATAGAT	
XMJ19-XXH-F	GGATCCCCGGGTACCGAGC	pXMJ19 linearized
XMJ19-XXH-R	TACTAGTCTCCTTCTTCTAGAGTCGACCTGCAGGCATG	
metX-Cg-XM-F	AGAAGAAGGAGACTAGTAATGCCCACCCTCGCGCCTT	pXM-*metX_Cg*
metX-Cg-XM-R	CTCGGTACCCGGGGATCCTTAGATGTAGAACTCGATGTAGGTCGAAGG	
metX^r_Lm-XM-F	AGAAGAAGGAGACTAGTAATGCCAACCTCCGAACAGAAC	pXM-*metX*^{r}*_Lm*
metX^r_Lm-XM-R	CTCGGTACCCGGGGATCCTTACAGGAACACGCCTTCATCG	
P_{trc}-metX_Lm-F	GAGTCTGAGGATCCTCTAGATTGACAATTAATCATCCGGCTCGTATAATGTGTGGAATT	pEC-*thrA*^{S345F}-P_{trc}-*metX*^{r}
P_{tac}-metX_Lm-F	GAGTCTGAGGATCCTCTAGATTGACAATTAATCATCGGCTCGTAT	pEC-*thrA*^{S345F}-P_{tac}-*metX*^{r}
$P_{NCgl1676}$-metX_Lm-F	GAGTCTGAGGATCCTCTAGAATGCGACAGTACTTTTCATTAAGCCTAA	pEC-*thrA*^{S345F}-$P_{NCgl1676}$-*metX*^{r}
P-metX_Lm-R	CCAAGCTTGCATGCCTGCAGTTACAG	

GAACACGCCTTCATCG

Table S4. The DNA sequence of promoter elements used in this study.

Promoter	Sequence (5’-3’)
P_{tuf}	GTAGGGTAAGTGGGGTAGCGGCTTGTTAGATATCTTGAAAT<br>CGGCTTTCAACAGCATTGATTTCGATGTATTTAGCTGGCCGT<br>TACCCTGCGAATGTCCACAGGGTAGCTGGTAGTTTGAAAATC<br>AACGCCGTTGCCCTTAGGATTCAGTAACTGGCACATTTTGTA<br>ATGCGCTAGATCTGTGTGCTCAGTCTTCCAGGCTGCTTATCA<br>CAGTGAAAGCAAAACCAATTCGTGGCTGCGAAAGTCGTAGC<br>CACCACGAAGTCCAGGAGGACATACA
P_{sod}	TAGCTGCCAATTATTCCGGGCTTGTGACCCGCTACCCGATAA<br>ATAGGTCGGCTGAAAAATTTCGTTGCAATATCAACAAAAAG<br>GCCTATCATTGGGAGGTGTCGCACCAAGTACTTTTGCGAAGC<br>GCCATCTGACGGATTTTCAAAAGATGTATATGCTCGGTGCGG<br>AAACCTACGAAAGGATTTTTTACCC
$P_{NCgl1676}$	ATGCGACAGTACTTTTCATTAAGCCTAAGAAAATTCCTTTAA<br>TTGACACTTAATTGACCAATAAGAGTCGATTAGATTGCATTA<br>TTAGGTAATCTAGTGATTTAATGGAGAATAAGAGCAACTGG<br>TGAAGAAAAGGCTTGATGAAAGAAGTTTTTTATCTAGCTAG<br>ATGTTCAATCACGAGCTTTAAGAAAGTATGTCAATAACTTTG<br>ACATAACCTAAACACAATAAATTATGTAGTATTATGTGACAC<br>TAAGTTATTACATTTATTATATGATTGGTTAGGACTATGGAC<br>ATG

References

1. Kirchner, O., Tauch, A. Tools for genetic engineering in the amino acid-producing bacterium *Corynebacterium glutamicum*. J Biotechnol. 2003; 104(1-3), 287-299.
2. Jakoby, M., Ngouoto-Nkili, C.E., Burkovski, A. Construction and application of new *Corynebacterium glutamicum* vectors. Biotechnol Techniques. 1999; 13(6), 437-441.
3. Li, N., Wang, M., Yu, S., Zhou, J. Optimization of CRISPR-Cas9 through promoter replacement and efficient production of L-homoserine in *Corynebacterium glutamicum*. Biotechnol J. 2021; 16(8), e2100093.
